# Clinical and immunological effects of adding cyclophosphamide to pomalidomide–dexamethasone in relapsed‐refractory multiple myeloma: The randomized MUKseven trial

**DOI:** 10.1111/bjh.70611

**Published:** 2026-06-16

**Authors:** Andrew Hall, James Croft, Katrina Walker, Kevin Boyd, Sadie Roberts, Amy Holroyd, Mamta Garg, Elsa Ferris, Gordon Cook, William E. Pierceall, Anjan Thakurta, Anita Gandhi, Charlotte Pawlyn, Sarah R. Brown, Martin Kaiser

**Affiliations:** ^1^ Clinical Trials Research Unit Leeds Institute of Clinical Trials Research, University of Leeds Leeds UK; ^2^ The Institute of Cancer Research London UK; ^3^ The Royal Marsden Hospital London UK; ^4^ Leicester Royal Infirmary Leicester UK; ^5^ National Institute for Health and Care Research Leeds Biomedical Research Centre Leeds UK; ^6^ Translational Development and Diagnostics Celgene Corporation Summit New Jersey USA

**Keywords:** cyclophosphamide, pomalidomide, relapsed‐refractory myeloma

## Abstract

There is an ongoing need for accessible combination therapies for patients with relapsed‐refractory multiple myeloma (RRMM), alongside a growing interest in understanding their immunological effects, particularly on T‐cell populations. Myeloma UK (MUK) MUKseven (NCT02406222) was an academic, UK multicentre randomized controlled, open‐label phase 2 trial. The planned sample size was 250 patients but recruitment was stopped early due to changes in standard of care. Between 2016 and 2018, 104 RRMM patients from 26 UK hospitals were recruited and randomized to receive cyclophosphamide, pomalidomide, and dexamethasone (CPd) or pomalidomide and dexamethasone (Pd) using minimization. Fifty‐four participants in each arm were analysed. The primary end‐point, progression‐free survival (PFS) was not met with median 6.9 months (95% Confidence Interval (CI): 5.7–10.4) for CPd versus 4.6 months for Pd (95% CI: 3.5–7.4), although not significant; reflecting underpowering from early closure. CPd showed a higher overall response rate, and toxicity was comparable to previously reported Pd regimens. Peripheral blood T‐cell analysis revealed stronger and more sustained enrichment of CD3+ T cells, HLA‐DR‐positive CD8+ and CD8+ effector memory cells in the CPd arm. Pretreatment CD4+ T‐cell levels were identified as a prognostic PFS marker. In summary, our results demonstrate significant effects of cycloaddition to Pd on response and T‐cell composition.

## INTRODUCTION

Treatment of patients with relapsed‐refractory myeloma (RRMM) remains a challenge.^1^ Although novel highly effective treatments such as T‐cell engaging (TCE) bispecific antibodies have shown impressive response rates and remission times, there is still a need for effective and affordable oral combination treatments.^2^ The combination of pomalidomide with cyclophosphamide and dexamethasone (CPd) gained refreshed interest in this context; however, insights into CPd effects beyond disease response are currently limited.^3^, ^4^, ^5^, ^6^ Characterization of combination therapy effects of CPd on the immune system over pomalidomide alone throughout different timepoints are of interest, as they could inform their potential future utility in the context of treatments such as TCEs, for which T cells form an essential part of the anti‐myeloma effect.^7^, ^8^ While some of the effects of pomalidomide on the immune system have been described, additional contributions of cyclophosphamide in a CPd combination are yet to be stringently isolated in a randomized clinical trial.^9^

The Myeloma UK (MUK) MUKseven trial was designed as a multi‐centre pomalidomide treatment optimization and biomarker discovery trial, randomizing patients to pomalidomide plus dexamethasone (Pd) or cyclophosphamide plus Pd (CPd) to investigate the efficacy of CPd and differential effects on immune cell populations and other molecular markers. We report here on the primary end‐point, comparing progression‐free survival (PFS) between Pd and CPd, as well as on a predefined comparison of their individual and differential effects on immune cell population dynamics alongside treatment.

## METHODS

MUKseven (NCT02406222) is a UK randomized, controlled, open‐label, parallel‐group, multicentre phase 2 trial to evaluate clinical efficacy of pomalidomide in combination with cyclophosphamide and dexamethasone (CPd), as compared to pomalidomide plus dexamethasone alone (Pd) in patients with RRMM. A secondary aim is to identify novel biomarkers of efficacy, reflected in a dense bio sampling strategy. The trial was designed as an independent academic trial, using UK National Health Service (NHS) research infrastructure, with the patient organization Myeloma UK serving as a design partner.

Patients were randomized 1:1 to receive either CPd (cyclophosphamide po 500 mg days 1, 8, 15; pomalidomide po 4 mg, days 1–21; dexamethasone po 40 mg [20 mg in patients ≥75 years], days 1, 8, 15, 22) or Pd (pomalidomide po 4 mg, days 1–21; dexamethasone po 40 mg [20 mg in patients ≥75 years], days 1, 8, 15, 22). Entry criteria stipulated that all patients had at least two prior lines of treatment and prior exposure to both lenalidomide (Len) and a proteasome inhibitor (PI) and refractoriness to either. Patients were allowed prior exposure to cyclophosphamide, due to frequent use in UK but not pomalidomide. Eligibility criteria were permissive, to include a real‐world RRMM population, including transfusions and growth factor support (See Supporting Information for full eligibility criteria). Patients continued treatment until progression. Randomization was performed centrally by the University of Leeds Clinical Trials Research Unit (CTRU), using minimization with a random element to balance for: age (<60 vs. 60–70 vs. ≥70), number of prior lines of therapy (≤3 vs. >3) and β2‑microglobulin (β2M) at randomization (<3.5 mg/L vs. 3.5–<5.5 mg/L vs. ≥5.5 mg/L).

As part of trial consent, patients agreed to provide a bone marrow (BM) and peripheral blood (PB) sample on cycle 1 day 1 (C1D1). Additional samples were taken at cycle 1 day 14 (C1D14), cycle 4 day 14 (C4D14) and at disease progression or treatment discontinuation (PD/dc). T‐cell subsets in PB were analysed using a commercially validated multi‐colour flow cytometry panel (detailed in Supporting Information including statistical analysis).

The full analysis set and safety analysis set were both defined as all registered patients on the trial that received at least one dose of pomalidomide. The primary end‐point was progression‐free survival (PFS) defined as the time from randomization to the first evidence of disease progression or death. Secondary end‐points included response to treatment, overall survival (OS), treatment compliance, safety, toxicity and sample compliance. Responses were defined according to International Myeloma Working Group (IMWG) guidelines.^10^ Safety and toxicity data were graded using National Cancer Institute Common Terminology Criteria for Adverse Events (NCI CTCAE) version 4.0.

A total of 250 patients (125 patients per arm) were required to demonstrate a 2‐month increase in median PFS with CPd compared to Pd, from 4.5 months to 6.5 months (hazard ratio [HR] 0.69) with 80% power and a 5% 2‐sided significance level, requiring 232 progression‐free survival (PFS) events to be observed. A log‐rank test was used to compare primary PFS between the treatment groups, stratifying for the minimization factors. PFS and OS curves were calculated using the Kaplan–Meier method. Response (≥partial response [PR]) was analysed using logistic regression, adjusting for minimization factors. Statistical analysis were performed using SAS (version 9.4).

The overall proportion of T cells and their subsets in relation to lymphocytes was summarized descriptively at C1D1, C1D14, C4D14 and PD/discontinuation for both treatment arms separately. Paired Wilcoxon signed‐rank tests were conducted on baseline (C1D1) and post‐baseline samples. Logistic regression was conducted for response by T‐cell subset adjusting for treatment and minimization factors. For PFS, a Cox's proportional hazards model adjusting for treatment and minimization factors, a local p‐spline model with 2 degrees of freedom for each T‐cell subset was fitted. A predictive model was also fitted with a treatment and T‐cell interaction term. Likelihood ratio tests were performed. Cut points were selected as the most significant relation using ‘survminer’ R package. All analyses were hypothesis generating without adjustment of *p*‐values. T‐cell statistical analysis was conducted in R (version 3.6.0).

## RESULTS

### Patient recruitment, trial closure and patient flow

One hundred and twenty‐three patients were registered to MUKseven across 26 UK sites between March 2016 and February 2018 and 104 patients of the originally planned 250 were randomized, 52 to each randomization arm (CPd and Pd). Recruitment to the trial was above target until December 2016, when pomalidomide became available to patients on the NHS. Beyond this date, recruitment continued, but at a rate below projections, which would have extended recruitment beyond financially sustainable and scientifically reasonable time frames. The independent data monitoring and ethics committee and trial steering committee therefore recommended early trial closure.

Final analysis data cut‐off to analyse all end‐points except OS was 24 October 2018, with median follow‐up of 13.4 months (95% CI: 12.0–17.5) and a median of 7 cycles of treatment received for the safety population (range 1–28). Sixteen patients were still on trial treatment at the time of the first data lock (median number of cycles: 19.5 cycles, range 8–28). The data cut‐off to analyse OS was 29 April 2020.

**FIGURE 1:**
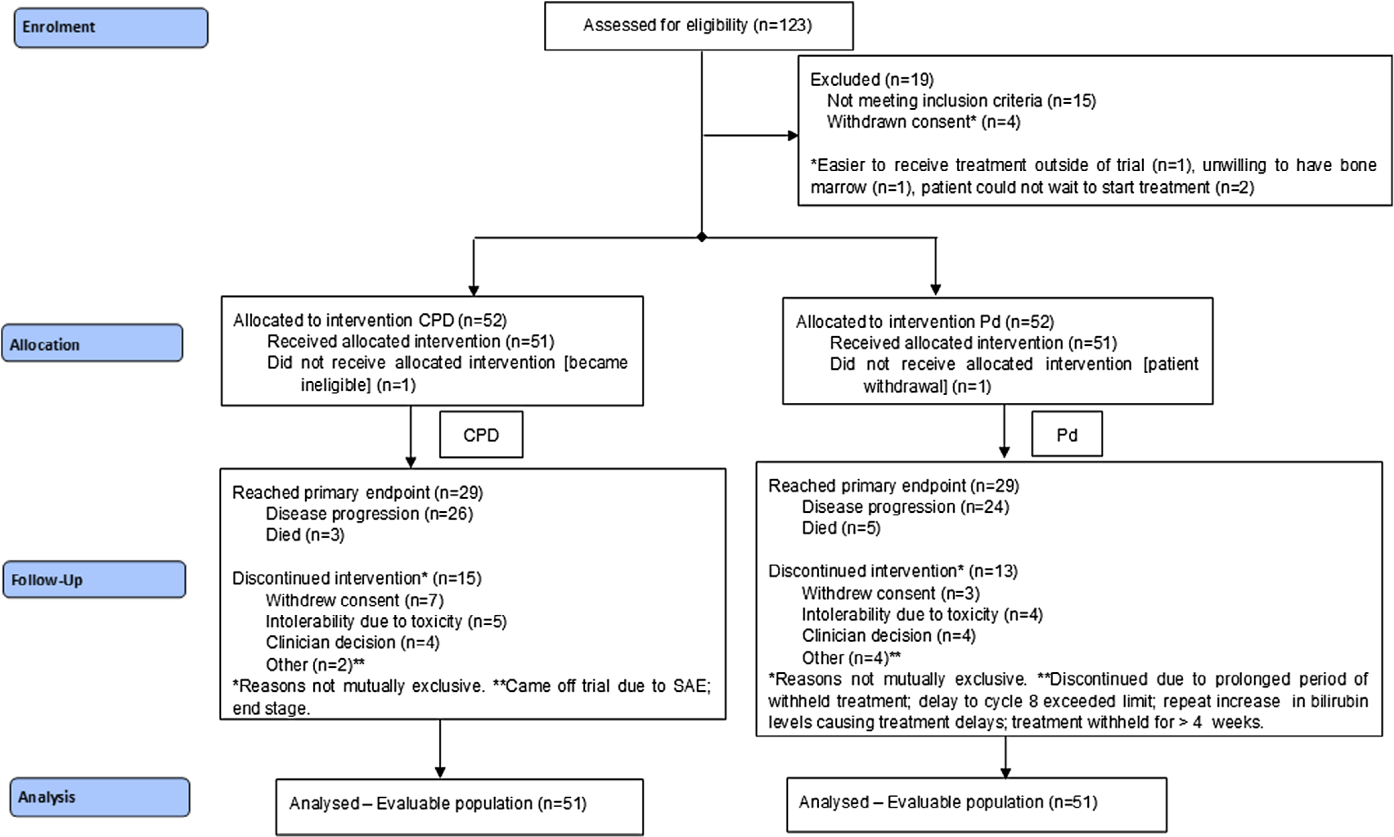
Consort diagram of the MUKseven trial. [Colour figure can be viewed at wileyonlinelibrary.com]

Patient flow, including randomization and details of withdrawals from the trial, are provided in Figure 1. Two patients were randomized but did not receive pomalidomide and were not included in the full analysis or safety analysis set. Baseline characteristics as well as types of and refractoriness to prior treatment were well balanced between groups (Table 1; Supporting Information). Previous cyclophosphamide treatment was seen in 94% of patients, 76.5% as part of two or more lines prior therapy (Supporting Information).

**TABLE 1:** Baseline characteristics of relapsed‐refractory myeloma patients in MUKseven overall and by treatment arm.

	CPd (*n* = 51)	Pd (*n* = 51)	Total (*n* = 102)
Age (years)			
Mean (s.d.)	68.1 (8.8)	67.9 (9.8)	68.0 (9.3)
Median (range)	69 (47, 88)	69 (42, 85)	69 (42, 88)
Age (categorized [years])			
<60	9 (17.6%)	8 (15.7%)	17 (16.7%)
60–69	18 (35.3%)	19 (37.3%)	37 (36.3%)
≥70	24 (47.1%)	24 (47.1%)	48 (47.1%)
Sex			
Male	32 (62.7%)	33 (64.7%)	65 (63.7%)
Female	19 (37.3%)	18 (35.3%)	37 (36.3%)
Time from diagnosis at randomization (years)			
Mean (s.d.)	6.2 (5.0)	5.9 (3.7)	6.0 (4.4)
Median (range)	5.0 (0.0, 26.0)	5.0 (0.0, 17.0)	5.0 (0.0, 26.0)
Number of prior lines of therapy			
2	11 (21.6%)	8 (15.7%)	19 (18.6%)
3	14 (27.5%)	20 (39.2%)	34 (33.3%)
4	10 (19.6%)	10 (19.6%)	20 (19.6%)
5 or more	16 (31.4%)	13 (25.5%)	29 (28.4%)
Beta‐2‐microglobulin			
Less than 3.5 mg/L	17 (33.3%)	17 (33.3%)	34 (33.3%)
3.5 mg/L—5.5 mg/L	18 (35.3%)	16 (31.4%)	34 (33.3%)
Greater than or equal to 5.5 mg/L	16 (31.4%)	18 (35.3%)	34 (33.3%)
Eastern Cooperative Oncology Group performance status			
0	20 (39.2%)	16 (31.4%)	36 (35.3%)
1	23 (45.1%)	27 (52.9%)	50 (49.0%)
2	8 (15.7%)	8 (15.7%)	16 (15.7%)
International Staging System at baseline			
Stage I	17 (33.3%)	17 (33.3%)	34 (33.3%)
Stage II	18 (35.3%)	16 (31.4%)	34 (33.3%)
Stage III	16 (31.4%)	18 (35.3%)	34 (33.3%)
Number of high‐risk abnormalities			
Single hit	23 (45.1%)	10 (19.6%)	33 (32.4%)
Double hit	2 (3.9%)	7 (13.7%)	9 (8.8%)
No lesions	10 (19.6%)	19 (37.3%)	29 (28.4%)
Missing/NA	16 (31.4%)	15 (29.4%)	31 (30.4%)
Platelet support prior to C1D1			
Yes	4 (7.8%)	7 (13.7%)	11 (10.8%)
No	47 (92.2%)	44 (86.3%)	91 (89.2%)
Neutrophil support prior to C1D1			
Yes	7 (13.7%)	9 (17.6%)	16 (15.7%)
No	44 (86.3%)	42 (82.4%)	86 (84.3%)

*Note*: Treatment arms are cyclophosphamide, pomalidomide, dexamethasone (CPd) and pomalidomide, dexamethasone (Pd).

### Survival analysis

**FIGURE 2:**
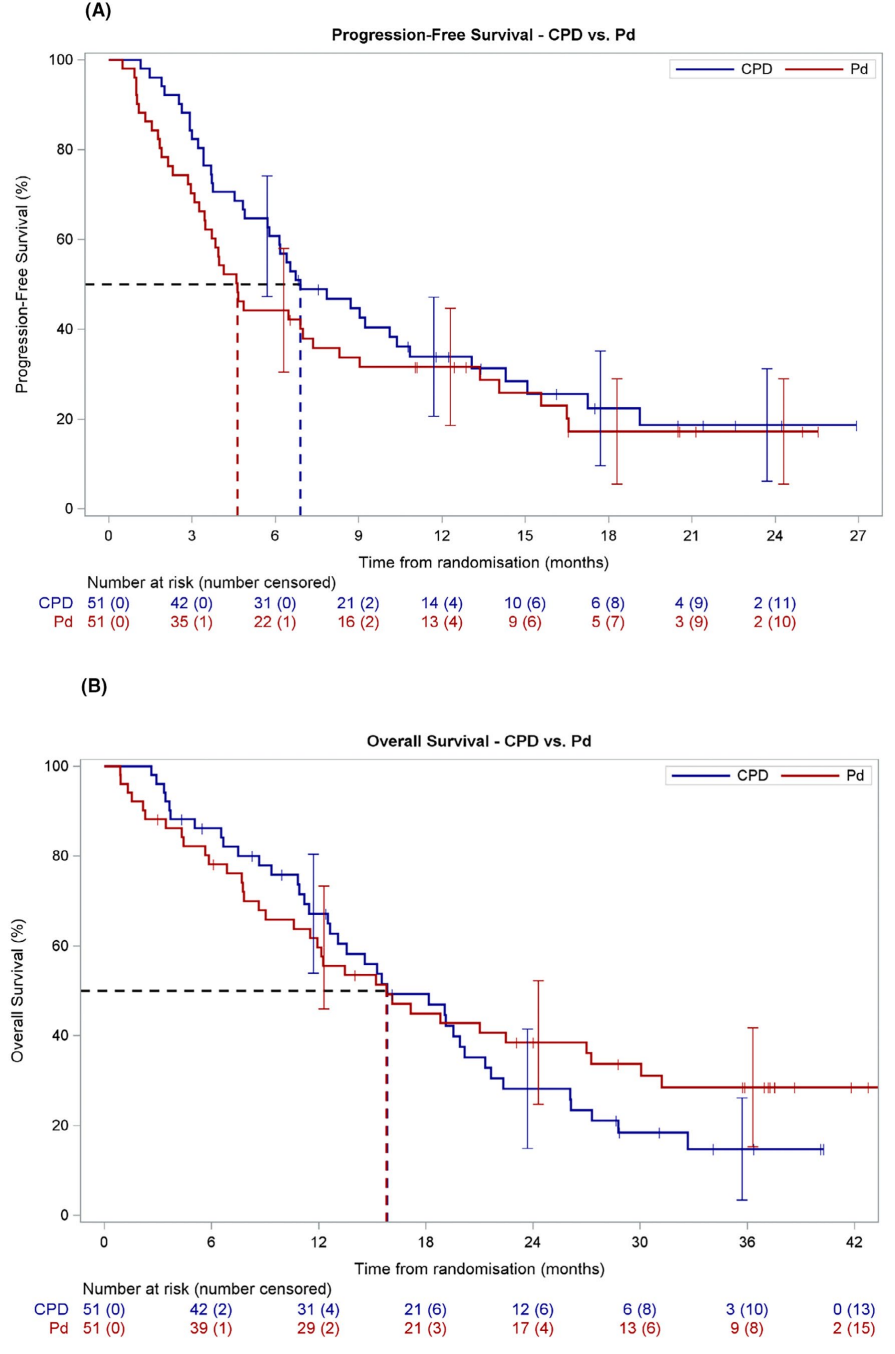
Survival analysis of MUKseven patients randomized to CPd versus Pd. (A) Progression‐free survival; (B) overall survival. [Colour figure can be viewed at wileyonlinelibrary.com]

In total, 77/102 patients had a progression event, with 64 of these events being disease progressions (CPd: 31, Pd: 33) and 13 being deaths (CPd: 7, Pd: 6). Median PFS was 6.9 months for CPd (95% CI: 5.7–10.4) versus 4.6 months for Pd (95% CI: 3.5–7.4) (Figure 2A). The primary log‐rank test was not significantly different (*p*‐value = 0.4). Six‐month PFS estimates were 60.8% for CPd (95% CI: 47.4%–74.2%) and 44.2% for Pd (95% CI: 30.4%–58.0%). PFS estimates at 12 months were 33.9% for CPd (95% CI: 20.6%–47.2%) and 31.6% for Pd (95% CI: 18.6%–44.7%). A Cox proportional hazards model adjusting for minimization factors estimated the HR for CPd versus Pd to 0.70 (95% CI: 0.44–1.11) (Supporting Information).

In total, 72/102 patients had died at the time of OS analysis (CPd: 38, Pd: 34). The most common primary cause of death was progressive disease, constituting 36.8% of CPd and 52.9% of Pd arm deaths.

Median OS was numerically identical at 15.8 months in both groups (CPd 95% CI: 12.6–20.2, Pd 95% CI: 11.5–27.0; *p*‐value = 0.9) (Figure 2B). The OS HR from the Cox proportional hazards model for CPd compared to Pd was 1.07 (95% CI: 0.66–1.73) (Supporting Information).

### Treatment response

The CPd arm had a higher overall response rate than Pd (ORR; PR or better), 70.6% (95% CI: 56.2–82.5%) compared to 47.1% (95% CI: 32.9–61.5%). Logistic regression analysis found an odds ratio of 3.49 (95% CI: 1.4–8.75) in favour of CPD (*p* = 0.006, Supporting Information).

Rates of very good partial response (≥VGPR) and complete response (CR) were 9.8% (95% CI: 3.3%–21.4%) and 9.8% (95% CI: 3.3%–21.4%) for CPd, respectively; while 17.6% (95% CI: 8.4%–30.9%) of patients on Pd achieved VGPR, none achieved CR. Of CPd arm patients, 9.8% (95% CI: 3.3%–21.4%) had a minimal response (MR) and 13.7% (95% CI: 5.7%–26.3%) stable disease (SD), as well as 29.4% (95% CI: 17.5%–43.8%) and 15.7% (95% CI: 7.0%–28.6%) of Pd patients respectively (Table 2).

**TABLE 2:** Maximum response: Serological response.

Maximum response	CPd (*n* = 51)	Pd (*n* = 51)	Total (*n* = 102)
CR	5 (9.8%)	0 (0.0%)	5 (4.9%)
VGPR	5 (9.8%)	9 (17.6%)	14 (13.7%)
PR	26 (51.0%)	15 (29.4%)	41 (40.2%)
MR	5 (9.8%)	8 (15.7%)	13 (12.7%)
SD or NC	7 (13.7%)	11 (21.6%)	18 (17.6%)
PD	3 (5.9%)	5 (9.8%)	8 (7.8%)
No maximum response^a^	0 (0.0%)	3 (5.9%)	3 (2.9%)

Abbreviations: CR, complete response; MR, maximum response; PD, progressive disease; PR, partial response; SD/NC, stable disease/no change; VGPR, very good partial response.

^a^ Patients who did not achieve a maximum response that is who withdrew or progressed prior to a response being observed, were summarized as ‘no maximum response’.

### Safety and deliverability

The most common higher‐grade toxicities experienced were thrombocytopenia and neutropenia: grade 4 occurrence was higher for both, for CPd‐treated patients (27.5% and 51.0%, respectively) than for Pd‐treated patients (15.7% and 19.6% respectively). In keeping, grade 3 or higher infections rate was 43.1% in CPd and 33.4% in Pd; however, the only infection‐related death occurred in the Pd arm. Overall, toxicity rates were comparable to previously reported Pd treatment regimens (Table 3). Adverse event categories were prespecified on the case report form. A total of 95 serious adverse events (SAEs) (77 serious adverse reactions [SARs], 18 non‐SAR SAEs) were reported on the CPd arm, while 75 SAEs (54 SARs, 21 non‐SAR SAEs) were reported on the Pd arm. Sixty‐three SAEs were caused by infection, as per CTCAE, on the CPd arm, and 49 on the Pd arm. The most common cause of non‐SAR SAEs on the trial was myeloma. Further details on adverse events, dose delays, reductions, omissions and mean dosing are provided in the Supporting Information.

**TABLE 3:** Adverse events.

Adverse event	CPd (*n* = 51)		Pd (*n* = 51)		Total (*n* = 102)	
	Any grade	Grade 3–4	Any grade	Grade 3–4	Any grade	Grade 3–4
Anaemia	38 (74.5%)	20 (39.2%)	39 (76.5%)	19 (37.3%)	77 (75.5%)	39 (38.2%)
Thrombocytopenia	33 (64.7%)	20 (39.2%)	24 (47.1%)	12 (23.5%)	57 (55.9%)	32 (31.4%)
Neutropenia	44 (86.3%)	38 (74.5%)	28 (54.9%)	18 (35.3%)	72 (70.6%)	56 (54.9%)
Diarrhoea	12 (23.5%)	2 (3.9%)	12 (23.5%)	1 (2.0%)	24 (23.5%)	3 (2.9%)
Constipation	15 (29.4%)	0 (0.0%)	8 (15.7%)	0 (0.0%)	23 (22.5%)	0 (0.0%)
Nausea	13 (25.5%)	1 (2.0%)	14 (27.5%)	1 (2.0%)	27 (26.5%)	2 (2.0%)
Vomiting	7 (13.7%)	0 (0.0%)	5 (9.8%)	0 (0.0%)	12 (11.8%)	0 (0.0%)
Sensory neuropathy	10 (19.6%)	0 (0.0%)	10 (19.6%)	0 (0.0%)	20 (19.6%)	0 (0.0%)
Peripheral neuropathy	18 (35.3%)	0 (0.0%)	15 (29.4%)	0 (0.0%)	33 (32.4%)	0 (0.0%)
Fatigue	35 (68.6%)	5 (9.8%)	35 (68.6%)	3 (5.9%)	70 (68.6%)	8 (7.8%)
Infection	35 (68.6%)	22 (43.1%)	38 (74.5%)	17 (33.3%)	73 (71.6%)	39 (38.2%)
Thromboembolic event	1 (2.0%)	1 (2.0%)	1 (2.0%)	0 (0.0%)	2 (2.0%)	1 (1.0%)
Oedema/ankle swelling	11 (21.6%)	0 (0.0%)	15 (29.4%)	0 (0.0%)	26 (25.5%)	0 (0.0%)
Rash	9 (17.6%)	2 (3.9%)	4 (7.8%)	0 (0.0%)	13 (12.7%)	2 (2.0%)
Cramp	11 (21.6%)	0 (0.0%)	10 (19.6%)	0 (0.0%)	21 (20.6%)	0 (0.0%)

### Treatment specific effects on T‐cell subsets

**FIGURE 3:**
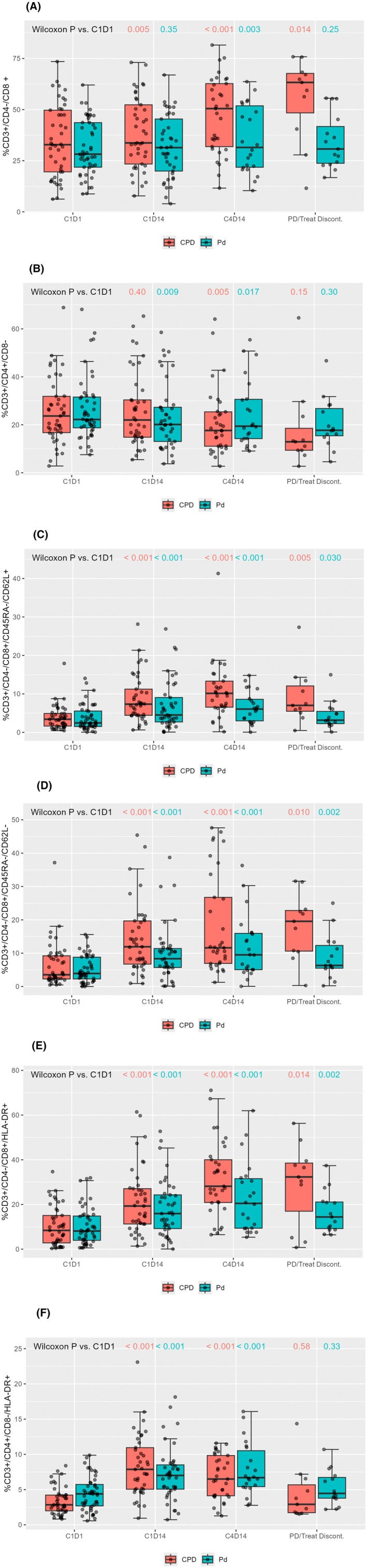
Treatment‐specific effects of CPd versus Pd on T‐cell subsets. Box and dot plots representing % of respective T‐cell population in relation to overall lymphocyte gate: (A) CD8+ T cells (%CD3+/CD4−/CD8+); (B) CD4+ T cells (%CD3+/CD4+/CD8−); (C) central memory CD8+ cells (%CD3+/CD4−/CD8+/CD45RA−/CD62L+); (D) effector memory CD8+ cells (%CD3+/CD4−/CD8+/CD45RA−/CD62L−); (E) activated CD8+ T cells (%CD3+/CD4−/CD8+/HLA‐DR+); (F) activated CD4+ T cells (%CD3+/CD4+/CD8−/HLA‐DR+). [Colour figure can be viewed at wileyonlinelibrary.com]

Flow cytometry data of PB T‐cell subsets was available for most patients at C1D1 (89%) and C1D14 (80%) and availability was comparable between arms and was lower at C4D14 (53%) and PD/dc (25%). Total T‐cell (CD3+) % relative to lymphocytes increased for patients in receipt of CPd at C1D14 and C4D14, but not for those receiving Pd alone (Supporting Information). Relative to total lymphocytes, there was a marked and significant increase in CD8+ population % at C4D14; this CD8+ % increase was not significantly different between CPd and Pd treated patients at C1D14, but at C4D14 increase in CD8+ % was markedly higher for CPd than for Pd compared to C1D1 (Figure 3A). CD4+ cell % reduced at C4D14 relative to baseline on CPd and Pd treatments (Figure 3B).

Importantly, within CD8+ sub‐populations, central memory (CD3+/CD4−/CD8+/CD45RA−/CD62L+) and CD8+ effector memory (CD3+/CD4−/CD8+/CD45RA−/CD62L−) % increased on both treatment arms and effector memory cells increased even significantly more with CPd than Pd (Figure 3C,D), while naïve CD8+ (CD3+/CD4−/CD8+/CD45RA+/CD62L+) and effector CD8+ (CD3+/CD4−/CD8+/CD45RA+/CD62L−) population % decreased with both CPd and Pd treatment (Supporting Information).

Relative to total lymphocytes, % of activated (HLA‐DR+) CD8+ T cells, as well as CD4+ T cells increased (Figure 3E,F). For CD8+ cells, this increase in mean % continued further to C4D14 and was enhanced by CPd over Pd alone; activated CD8+ cells for patients in receipt of CPd constituted a median of 28.1% (range 6.5% to 71.0%) of the overall lymphocyte pool.

There was also an increase in % of proliferating (Ki67+) T‐cell sub‐populations on treatment, both CD4+ and CD8+ (Supporting Information). Proliferating CD8+ cells constituted a median of 9.0% (range 1.3 to 44.0) of all lymphocytes at C4D14 for CPd; the majority of these proliferating CD8+ cells also showed markers of activation (Supporting Information).

### Pretreatment T cells and their effect on response and prognosis

We also investigated whether T‐cell composition immediately pretreatment was associated with response to CPd or Pd. Low CD4+ cell count % at baseline was significantly associated with a low probability of reaching PR with treatment across CPd and Pd (*p* = 0.02). There was no association between CD8+ or overall T‐cell (CD3+) % and response (*p* = 0.25, *p* = 0.68 respectively). Pretreatment C1D1 CD4+ % cell counts were significantly associated with PFS across both treatment arms (*p* = 0.02). Patients with a lower CD4+ baseline % count had an increasingly worse prognosis compared to those with a higher count. Sensitivity analyses using a treatment‐CD4% interaction term found no evidence of effect modification, indicating that baseline CD4% was prognostic rather than predictive.

**FIGURE 4:**
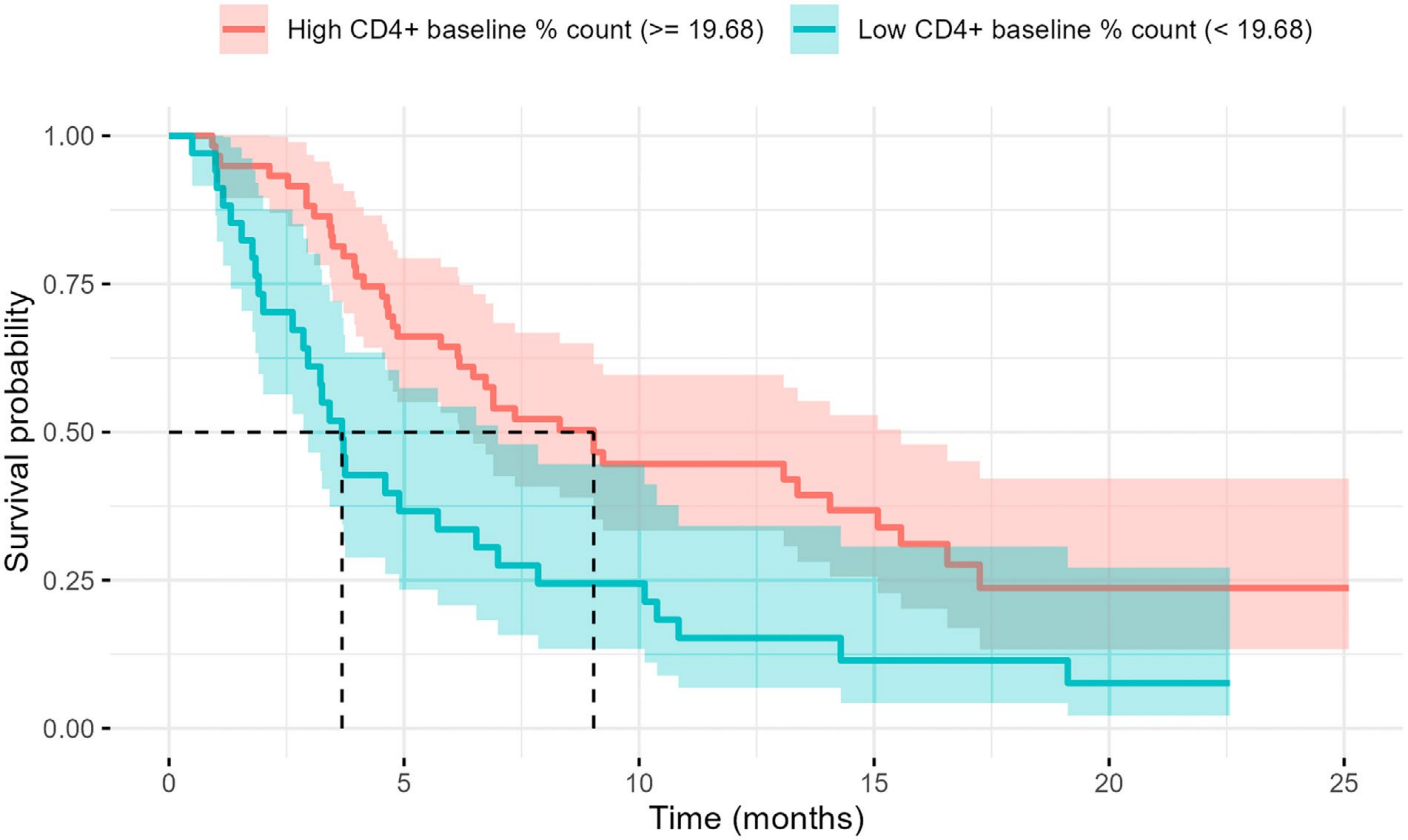
Pretreatment C1D1 CD4+ % cell counts associated with progression‐free survival. An optimal binary threshold of 19.68 corresponded with Median PFS of 3.7 months for those with a low CD4+ baseline % count (95% CI: 2.9–7.0) versus 9.0 months for high % count (95% CI: 6.2–15.6). [Colour figure can be viewed at wileyonlinelibrary.com]

Further analysis found a strong log‐linear relationship between CD4+ % and relative progression rate (Supporting Information). An optimal binary threshold of 19.7% CD4+ cells was used to define a low and high CD4+ % group of patients (Figure 4). Median PFS was 3.7 months for those with a low CD4+ baseline % count (95% CI: 2.9–7.0) versus 9.0 months for patients with a high % count (95% CI: 6.2–15.6). Low baseline CD4+ central memory (%CD3+/CD4+/CD8−/CD45RA−/CD62L+) and effector memory (%CD3+/CD4+/CD8−/CD45RA−/CD62L−) cell counts also linked to poorer outcome (*p* = 0.03, *p* = 0.12 respectively).

## DISCUSSION

MUKseven was specifically designed, with the patient organization Myeloma UK, as a treatment optimization trial, exploring an affordable and accessible combination treatment. The trial sought to inform practice outside and in addition to, the standard regulatory pathway; and as a biomarker discovery trial, with a dense peripheral blood and bone marrow sampling schedule, to explore the biological impacts of the affordable CPd combination over the licensed Pd combination alone. CPd exhibited a nominally longer progression‐free survival (PFS) compared to Pd; although not achieving statistical significance, this result is inconclusive due to the trial being underpowered due to the curtailment of recruitment for MUKseven. With the observed 77 PFS events, there was 36% power to detect an improvement in 2 months median PFS as specified in the sample size. Response rates were also higher with CPd than with Pd.

Two positive trials comparing CPd and Pd have now been reported.^3^, ^11^ Baz et al. showed a 5‐month improvement in PFS by addition of cyclophosphamide, with 70 patients included and randomized between the two arms.^3^ The trial also included a dose‐finding run‐in phase, which determined 400 mg cyclo weekly po as the optimal dose. MUKseven adopted the standard 500 mg cyclo weekly dose that was widely used with cyclophosphamide lenalidomide and dexamethasone (CRd) in the United Kingdom at the time and safety and dose intensity were closely monitored. Monitoring of dose reductions was a prespecified aim in MUKseven and cyclo required reduction in 28 (54.9%) patients. There was a higher number of SAEs in the CPd arm, but no excess of treatment‐related deaths. A recent publication by the Korean myeloma study group also demonstrated a significantly longer PFS with CPd over Pd in a randomized trial.^11^ In the context of this study being underpowered due to having to stop recruitment early, it is important to note that results from MUKseven should not be interpreted as a negative finding. Results are in our view consistent with the external evidence in favour of CPd, making CPd a potential treatment option in resource‐limited settings or when other treatment targets have been exhausted.

The biomarker discovery results of MUKseven are informative despite curtailed recruitment, with a high proportion of recruited patients having completed longitudinal immune cell profiling at baseline and on C1D14, enhancing the representativeness of the results. Marked effects of Pd on T‐cell composition were confirmed with high certainty, including the increase of CD8+ T cells and particularly that of HLA‐DR‐positive activated CD4+ and CD8+ populations. In addition, our results demonstrate a specific effect of cyclophosphamide addition, with a sustained increase in CD3+ T‐cell % overall, and specifically, an increase in activated CD8+ cells and an increase in CD8+ effector memory cells in CPd treated patients. The design of the trial and the analysis means that this enhancing effect of cyclophosphamide can be isolated: pomalidomide and dexamethasone doses administered were similar across randomization arms, and baseline interpatient variability in T‐cell composition was accounted for.

Interestingly, recent work identified the presence of a high fraction of CD8+ T effector memory cells to be associated with response to anti‑B‑cell maturation antigen (BCMA) TCEs.^12^, ^13^, ^14^, ^15^ The optimal use of TCEs is still being explored, including pretreatment debulking strategies to enhance response rates. Our results suggest the potential utility of CPd for debulking and enhancing immune cell composition prior to TCE treatment or as a potential for bridging therapy after lymphocyte collection in the context of chimeric antigen receptor T‑cell (CAR‐T), in case other treatment options are not applicable. However, importantly, cyclophosphamide use as in CPd should be avoided in patients potentially still scheduled to undergo lymphocyte collection for CAR‐T cell therapy in the intermediate future, in accordance with National Comprehensive Cancer Network (NCCN) guidelines.

We also demonstrate a striking quantitative relationship between lower CD4+ T‐cell counts pretreatment and shorter PFS, across Pd and CPd arms. Pomalidomide has been described to stimulate high levels of interleukin‑2 (IL‐2) production predominantly in CD4+ cells.^16^ Interestingly, CD4+ % remained unchanged with start of Pd or CPd treatment in this study, although the percentage of activated CD4+ cells increased, potentially underpinning that the pretreatment CD4+ count may be important for later treatment effects.

Pomalidomide‐induced IL‐2 transcription in T cells is mediated by Aiolos degradation and Aiolos has been demonstrated to repress CD4+ cytotoxic programming.^17^ In vivo work found that depletion of CD4+ cells, but not of other cell types such as CD8+ or natural killer (NK) cells, to abrogate the anti‐myeloma activity of lenalidomide in immunocompetent mice bearing 5TGM1 murine myeloma cells.^18^ Low levels of CD4+ cells may therefore limit the early immunogenic anti‐myeloma pomalidomide effects. As induction of IL‐2 was predominantly observed with second generation immunomodulatory drugs (IMiDs) lenalidomide and pomalidomide, but barely with thalidomide, it might be of particular interest to study the prognostic significance of CD4+ baseline counts also with newer cereblon binding IMiDs, such as iberdomide and mezigdomide.^19^

In addition to the effects measured in this study, other immune cell compartments that have been previously reported as being relevant to cyclophosphamide as well as pomalidomide activity, including NK cells, Tregs or myeloid‐derived suppressor cells. These cell compartments were not assessed here, constituting a potential limitation of this study.

In summary, our results demonstrate an increased response rate with CPd over Pd and a marked and sustained enrichment of T‐cell populations with CPd, with potential future implications for the utility of CPd in the context of T‐cell‐based treatments.

## AUTHOR CONTRIBUTIONS

**Andrew Hall:** Formal analysis; conceptualization; methodology; writing – original draft; writing – review and editing; validation; visualization. **William E. Pierceall:** Resources. **Kevin Boyd:** Investigation. **Mamta Garg:** Investigation. **Anjan Thakurta:** Investigation. **Anita Gandhi:** Resources. **Gordon Cook:** Investigation. **Elsa Ferris:** Investigation. **James Croft:** Investigation. **Sarah R. Brown:** Conceptualization; formal analysis; writing – review and editing; methodology; supervision. **Sadie Roberts:** Funding acquisition; project administration; data curation; validation. **Charlotte Pawlyn:** Investigation. **Katrina Walker:** Formal analysis. **Amy Holroyd:** Investigation. **Martin Kaiser:** Conceptualization; investigation; writing – review and editing; writing – original draft; supervision.

## FUNDING INFORMATION

This trial was developed through the Myeloma UK Early Phase Clinical Trials Network and funded by Myeloma UK and Celgene. Celgene approved the design of the study but had no input in the data collection, data analysis, data interpretation or writing of the report, as this is a fully academically sponsored trial. The molecular risk model was developed with support by Myeloma UK and the NIHR Biomedical Research Centre at The Institute of Cancer Research and The Royal Marsden Hospital.

## CONFLICT OF INTEREST STATEMENT

Martin Kaiser: Honoraria: Amgen, BMS/Celgene, Janssen, Takeda; Consulting or advisory role: Amgen, AbbVie, BMS/Celgene, GSK, Janssen, Pfizer, Regeneron, Seattle Genetics, Takeda; Research funding: BMS/Celgene, Janssen; Travel, accommodation, expenses: BMS/Celgene, Janssen, Takeda.

Andrew Hall: Research funding: Janssen (Inst), Bristol Myers Squibb/Celgene (Inst). Sarah R Brown: Research Funding: AstraZeneca (Inst), Janssen (Inst), Bristol Myers Squibb/Celgene (Inst), Adlai Nortye (Inst). Charlotte Pawlyn: Honoraria: Abbvie, Amgen, Janssen, Celgene/BMS, Menarini Stemline, Sanofi. Consulting or advisory role: Abbvie, Amgen, Janssen, Celgene/BMS, Sanofi, iTEOS, Pfizer. Gordon Cook: Honoraria: Amgen, Janssen, Takeda; Consulting or advisory role: Amgen, AbbVie, BMS/Celgene, GSK, Janssen, Pfizer, Takeda; Research funding: Janssen, BMS/Celgene, Takeda. All other authors report no conflict of interest.

## ETHICS STATEMENT

This study was conducted in accordance with the Declaration of Helsinki and received national research ethics approval from the NHS National Research Ethics Service East of England, Cambridge (REC Number: 15/EE/0421).

## PATIENT CONSENT STATEMENT

Written informed consent was obtained from all individual participants included in the study.

## TRIAL REGISTRATION

This study was registered on the International Standard Randomized Controlled Trial Number registry, number ISRCTN24593488.

## Supporting information


Data S1.

## Data Availability

Any requests for individual participant data will be reviewed by the trial management group in the first instance and considered following completion of all primary and secondary end‐point analyses of the trial. Only requests that have a methodologically sound proposal and whose proposed use of the data has been approved by the independent trial steering committee will be considered. Proposals should be directed to the corresponding author in the first instance; to gain access, data requestors will need to sign a data access agreement.
