# Supplementary material for: Clinical and immunological effects of adding cyclophosphamide to pomalidomide–dexamethasone in relapsed‐refractory multiple myeloma: The randomized MUKseven trial
Source: Br J Haematol. 2026 Jun 16;209(2):557–68. doi: 10.1111/bjh.70611 (PMC13461968; doi:10.1111/bjh.70611)
Supplement: Supplementary file 1 [file BJH-209-557-s001.docx]

Supplementary Material

# Contents

[Contents	1](#_Toc204600105)

[1.	Trial flow diagram	4](#_Toc204600106)

[2.	Trial Registration	5](#_Toc204600107)

[3.	Inclusion Criteria	5](#_Toc204600108)

[4.	Exclusion Criteria	6](#_Toc204600109)

[5.	T cell standardized multi-color flow cytometry panel	7](#_Toc204600110)

[6.	Baseline characteristics	7](#_Toc204600111)

[6.1.	Baseline characteristics - time since relapse, first ASCT and last anti-myeloma treatment at randomization	7](#_Toc204600112)

[6.2.	Baseline summaries of previous exposure to cyclophosphamide and refractoriness to treatment	8](#_Toc204600113)

[6.3.	Previous treatment: Carfilzomib, Bortezomib and Lenalidomide	9](#_Toc204600114)

[7.	Progression free Survival	9](#_Toc204600115)

[7.1.	Hazard Ratios for progression-free survival using the Cox model, with corresponding 95% confidence intervals and P-values	9](#_Toc204600116)

[8.	Overall Survival	10](#_Toc204600117)

[8.1.	Parameter Estimates, standard errors and Hazard Ratios for overall survival using the Cox model, with corresponding 95% confidence intervals and P-values	10](#_Toc204600118)

[9.	Treatment Response	10](#_Toc204600119)

[9.1.	Parameter estimates, standard errors and odds ratios of response to treatment, with corresponding 95% confidence intervals and p-values	10](#_Toc204600120)

[10.	Safety	10](#_Toc204600121)

[10.1.	Number of participants with one or more SAE reported (mutually exclusive)	11](#_Toc204600122)

[10.2.	Summary statistics of the number of SAEs reported	11](#_Toc204600123)

[10.3.	Suspected relationship to trial medications for all reported SAEs	11](#_Toc204600124)

[10.4.	All SAEs by MedDRA system organ class (currently mutually exclusive)	12](#_Toc204600125)

[10.5.	SAEs suspected to be related to trial medications by MedDRA system organ class	12](#_Toc204600126)

[11.	Adverse Events	13](#_Toc204600127)

[11.1.	Maximum grade AE experienced for each patient not reported as pre-defined term	13](#_Toc204600128)

[11.2.	Other Adverse Reactions (grade 3+) with grade 4 and 5 events highlighted	13](#_Toc204600129)

[12.	Deliverability	17](#_Toc204600130)

[12.1.	Dose modifications.	18](#_Toc204600131)

[13.	Treatment specific effects on T-cell subsets	19](#_Toc204600132)

[13.1.	Total T cells	19](#_Toc204600133)

[13.2.	CD4+ T cells	20](#_Toc204600134)

[13.3.	CD8+ T cells	20](#_Toc204600135)

[13.4.	Proliferating T cells	21](#_Toc204600136)

[13.5.	Proliferating CD4+ T cells	21](#_Toc204600137)

[13.6.	Proliferating CD8+ T cells (include)	23](#_Toc204600138)

[13.7.	Naïve or effector CD4+ T cells	24](#_Toc204600139)

[13.8.	Naïve CD4+ T cells	24](#_Toc204600140)

[13.9.	Effector CD4+ T cells	25](#_Toc204600141)

[13.10.	Central memory CD4+ T cells	25](#_Toc204600142)

[13.11.	Effector memory CD4+ T cells	26](#_Toc204600143)

[13.12.	Naïve or effector CD8 + T cells	26](#_Toc204600144)

[13.13.	Naïve CD8+ T cells	27](#_Toc204600145)

[13.14.	Effector CD8+ T cells	28](#_Toc204600146)

[13.15.	Central memory CD8+ T cells	29](#_Toc204600147)

[13.16.	Effector memory CD8+ T cells	29](#_Toc204600148)

[13.17.	Proliferating naïve or effector CD4+ T cells	30](#_Toc204600149)

[13.18.	Proliferating naïve CD4+ T cells	30](#_Toc204600150)

[13.19.	Proliferating effector CD4+ T cells	31](#_Toc204600151)

[13.20.	Proliferating central memory CD4+ T cells	31](#_Toc204600152)

[13.21.	Proliferating effector memory CD4+ T cells	32](#_Toc204600153)

[13.22.	Proliferating naïve or effector CD8+ T cells	32](#_Toc204600154)

[13.23.	Proliferating naïve CD8+ T cells	33](#_Toc204600155)

[13.24.	Proliferating effector CD8+ T cells	33](#_Toc204600156)

[13.25.	Proliferating central memory CD8+ T cells	34](#_Toc204600157)

[13.26.	Proliferating effector memory CD8+ T cells	34](#_Toc204600158)

[13.27.	Activated CD4+ T cells	35](#_Toc204600159)

[13.28.	Activated CD8+ T cells	35](#_Toc204600160)

[13.29.	Proliferating activated CD8+ T cells	36](#_Toc204600161)

[13.30.	Proliferating activated CD4+ T cells	37](#_Toc204600162)

[13.31.	Ratio CD4+:CD8+ T cells	38](#_Toc204600163)

[14.	Pre-treatment T-cells and their effect on response and prognosis	38](#_Toc204600164)

[14.1.	Total T cells (C1D1) response analysis	38](#_Toc204600165)

[14.2.	CD4+ T cells (C1D1) Response analysis	38](#_Toc204600166)

[14.3.	CD8+ T cells (C1D1) Response analysis	39](#_Toc204600167)

[14.4.	CD4+ T cells Prognostic PFS analysis	39](#_Toc204600168)

## 1. Trial flow diagram

**Consent**

Written informed consent must be obtained prior to commencement of trial specific assessments. **Bone marrow and blood samples must be obtained**

**Registration**

Registration will be administered by the web using an automated 24 hour system. This will provide a patient trial number.

<https://lictr.leeds.ac.uk/webrand/>

**Central Lab Samples & Confirmation from Lab**

Samples must be taken and confirmation of receipt from the laboratories received before confirming the patient is deemed eligible

**Treatment Period**

Participants will receive treatment with either pomalidomide and dexamethasone or pomalidomide, cyclophosphamide and dexamethasone until disease progression

Bone marrows must be done at Cycle 1 & 4 and disease progression

**Eligibility**

Eligibility must be confirmed by CTRU. If the patient is not eligible complete Eligibility Checklist and send to CTRU

**Randomisation**

CTRU will confirm the patient is eligible by email to allow the patient to be randomised using the 24 hour system. https://lictr.leeds.ac.uk/webrand/Confirmation will be sent to the trials team and pharmacy

## 2. Trial Registration

This study was registered on the International Standard Randomized Controlled Trial Number registry, number ISRCTN24593488. The trial received national research ethics approval from the East of England – Cambridge East Research Ethics Committee (REC Number: 15/EE/0421), the institutional review boards of the participating centers, and the competent regulatory authority (Medicines and Healthcare Products Regulatory Agency, London, UK), and was undertaken according to the Declaration of Helsinki and the principles of Good Clinical Practice. All patients provided written informed consent.

## 3. Inclusion Criteria

***Participants must meet all of the following inclusion criteria to be eligible to enrol in this trial. Eligibility waivers will not be granted under any circumstances in this trial.***

- Diagnosed with symptomatic multiple myeloma (according to IMWG 2014 criteria)(1) and have measurable disease
- Participants must require therapy for relapsed and/or refractory disease
- Participants must have received ≥ 2 treatment lines of anti-myeloma therapy (induction therapy followed by ASCT and consolidation/maintenance will be considered as one line).
- Participants must have received prior treatment with both lenalidomide and a proteasome inhibitor, either as single agents or in combination regimens
- All participants must have failed treatment with either lenalidomide and a proteasome inhibitor in one of the following ways:

1) Documented progressive disease on or within 60 days of completing treatment with lenalidomide and/or a proteasome inhibitor; **or**

2) In case of prior response [≥ partial response (PR)] to lenalidomide or a proteasome inhibitor, participants must have relapsed within 6 months after stopping treatment with lenalidomide and/or a proteasome inhibitor-containing regimens; **or**

3) Participants who have not had a ≥ minimal response (MR) despite either receiving at least 4 cycles of treatment, or who have developed intolerance/toxicity after a minimum of two cycles of lenalidomide- and/or a proteasome inhibitor-containing regimen

- Patients must have received adequate prior alkylator therapy in one of the following ways

a. As part of a stem cell transplant; **or**
b. A minimum of 4 consecutive cycles of an alkylator based therapy; **or**
c. Progression on treatment with an alkylator; provided that the participant received at least 2 cycles of an alkylator-containing therapy.

- Life expectancy of at least 3 months
- Eastern Cooperative Oncology Group (ECOG) performance status score of 0, 1, or 2
- Required laboratory values at registration:
  - Absolute neutrophil count ≥ 1.0 x10^9^ /L (growth factor support is permitted)
  - Platelet count ≥ 30 x 10^9^/L (platelet transfusion is permitted)
  - Creatinine clearance > 30 mL/min (using Cockcroft Gault formula)
  - Corrected serum calcium ≤ 3.5 mmol/L
  - Haemoglobin ≥ 80 g/L (blood transfusion support is permitted)
  - Aspartate aminotransferase (AST) or Alanine aminotransferase (ALT) < 3 times Upper Limit of Normal (ULN)
  - Serum total bilirubin < 17µmol/l
- Participants must consent to provide the bone marrow samples specified at screening and throughout the trial, in order to enter the trial. Confirmation of receipt of the sample from the lab must be received before treatment commences.
- Able to give informed consent and willing to follow trial protocol
- Aged over 18 or over
- Females of childbearing potential (FCBP) must agree to utilise two reliable forms of contraception simultaneously or practice complete abstinence for at least for 28 days prior to starting trial treatment, during the trial and for at least 28 days after trial treatment discontinuation, and even in case of dose interruption, and must agree to regular pregnancy testing during this timeframe.
- Females must agree to abstain from breastfeeding during trial participation and 28 days after trial drug discontinuation
- Males must agree to use a latex condom during any sexual contact with FCBP during the trial, including during any dose interruptions and for 28 days following discontinuation from this trial even if he has undergone a successful vasectomy
- Males must also agree to refrain from donating semen or sperm while on pomalidomide, including during any dose interruptions and for 28 days after discontinuation from this trial
- All participants must agree to refrain from donation blood while on trial drug, including during dose interruptions and for 28 days after discontinuation from this trial

## 4. Exclusion Criteria

***Participants meeting any of the following exclusion criteria are not eligible to enrol in this trial. Eligibility waivers will not be granted under any circumstances in this trial.***

- Previous therapy with pomalidomide
- Hypersensitivity to thalidomide, lenalidomide, cyclophosphamide or dexamethasone
- Participants with non-secretory multiple myeloma
- Peripheral neuropathy ≥ Grade 3
- Participants who have received an allogeneic bone marrow or allogeneic peripheral blood stem cell transplant
- Previous anti-tumour therapies including investigational medicinal products at any dose within 28 days before the start of protocol treatment (or 5 half-lives, whichever is longer). A pulse of dexamethasone may be given 14 days before the start of protocol treatment. Bisphosphonates for bone disease and radiotherapy for palliative intent are permitted.
- Participants with any of the following

a. Uncontrolled congestive heart failure
b. Myocardial infarction within 12 months prior to starting trial treatment
c. Unstable or poorly controlled angina pectoris, including Prinzmetal variant angina pectoris.

- Participants with gastrointestinal disease that may significantly alter absorption of pomalidomide
- Participants with a history of other malignancies within 5 years before the date of study entry (exceptions are squamous and basal cell carcinomas of the skin, carcinoma in situ of the cervix or breast, or other non-invasive lesion that is considered cured with minimal risk of recurrence within 5 years).
- Participants unable or unwilling to undergo antithrombotic prophylactic treatment
- Pregnant or breastfeeding females
- Participants known to be seropositive for HIV, or active infectious hepatitis A, B or C. Participants that test positive for hepatitis B without active infection will not be excluded but caution and close monitoring will be required.
- Participants for any reason unwilling or cannot  consent to follow the Pomalidomide pregnancy prevention programme
- Any condition including the presence of laboratory abnormalities, which places the participant at unacceptable risk if they were to participate in the trial

## 5. T cell standardized multi-color flow cytometry panel

Fresh peripheral blood (PB) T-cell populations were profiled using a commercially validated multicolor flow cytometry (MFC) assay performed at Covance/Labcorp (Mechelen, Belgium) who were blinded to clinical data. The assay was run on a BD FACSCanto II cytometer, and data were analysed using FlowJo software according to Covance’s validated standard operating procedures.

The panel was designed to characterise baseline and pharmacodynamic changes in cytotoxic, naïve, memory, and activated/proliferating T-cell subsets. Markers included CD3, CD4, CD8, CD45RA, CD62L, HLA-DR, and Ki67, together with the corresponding commercial isotype controls. T-cell sub-populations were defined and their respective % of total lymphocyte population used for downstream analyses. The validated antibody/fluorochrome panel provided by Covance is shown below.

| Marker / Fluorochrome | T cell P Panel |
| --- | --- |
| Tube ID | T cell P |
| FITC | CD45RO (isotype) |
| PE | CD62L (isotype) |
| PerCP | HLA DR (isotype) |
| APC | CD45RA |
| AF700 | CD8 |
| V450 | CD4 |
| PacOrange | CD45 |
| Biotin Qdot | CD3 |

## 6. Baseline characteristics

### 6.1. Baseline characteristics - time since relapse, first ASCT and last anti-myeloma treatment at randomization

|  | **CPD (n=51)** | **Pd (n=51)** | **Total (n=102)** |
| --- | --- | --- | --- |
| **Time since most recent relapse, partial dates incorporated (months)*** | | | |
| Mean (s.d.) | 14.5 (39.9) | 8.1 (7.2) | 11.3 (28.7) |
| Median (range) | 4.6 (1.8, 284.3) | 5.0 (1.3, 32.8) | 4.8 (1.3, 284.3) |
| Missing | 0 | 0 | 0 |
| Number included | 51 | 51 | 102 |
| **Time since first ASCT, partial dates incorporated (years)*** | | | |
| Mean (s.d.) | 6.2 (4.0) | 5.5 (4.1) | 5.9 (4.1) |
| Median (range) | 4.9 (0.6, 17.5) | 4.4 (-0.1, 17.2) | 4.6 (-0.1, 17.5) |
| Missing | 21 | 17 | 38 |
| Number included | 30 | 34 | 64 |
| **Time since last dose of anti-myeloma treatment, no partial dates (months)** | | | |
| Mean (s.d.) | 10.9 (17.1) | 5.9 (4.6) | 8.4 (12.8) |
| Median (range) | 4.0 (1.6, 77.3) | 4.1 (0.7, 19.9) | 4.1 (0.7, 77.3) |
| Missing | 9 | 10 | 19 |
| Number included | 42 | 41 | 83 |
| **Time since last dose of anti-myeloma treatment, partial dates incorporated (months)*** | | | |
| Mean (s.d.) | 13.6 (25.5) | 7.2 (5.8) | 10.4 (18.7) |
| Median (range) | 4.3 (1.6, 153.3) | 5.3 (0.7, 24.1) | 4.5 (0.7, 153.3) |
| Missing | 0 | 0 | 0 |
| Number included | 51 | 51 | 102 |

Where dates are given as partial, missing days and months are set as 15 and 06 respectively.

### 6.2. Baseline summaries of previous exposure to cyclophosphamide and refractoriness to treatment

|  | **CPD (n=51)** | **Pd (n=51)** | **Total (n=102)** |
| --- | --- | --- | --- |
| **Total previous lines of cyclophosphamide** | | | |
| 0 | 3 (5.9%) | 3 (5.9%) | 6 (5.9%) |
| 1 | 8 (15.7%) | 10 (19.6%) | 18 (17.6%) |
| 2 | 27 (52.9%) | 24 (47.1%) | 51 (50.0%) |
| 3 | 7 (13.7%) | 12 (23.5%) | 19 (18.6%) |
| 4 | 6 (11.8%) | 1 (2.0%) | 7 (6.9%) |
| 5 | 0 (0.0%) | 1 (2.0%) | 1 (1.0%) |
| **Total** | **51 (100%)** | **51 (100%)** | **102 (100%)** |
| **Refractory to treatment*** | | | |
| Carfilzomib | 1 (20.0%) | 0 (0.0%) | 1 (6.7%) |
| Bortezomib | 3 (60.0%) | 7 (70.0%) | 10 (66.7%) |
| Lenalidomide | 1 (20.0%) | 3 (30.0%) | 4 (26.7%) |
| **Total** | **5 (100%)** | **10 (100%)** | **15 (100%)** |

*This includes all patients that listed ‘Refractory’ as a reason for discontinuing this treatment.

### 6.3. Previous treatment: Carfilzomib, Bortezomib and Lenalidomide

|  | **CPD (n=51)** | **Pd (n=51)** | **Total (n=102)** |
| --- | --- | --- | --- |
| **Carfilzomib** |  |  |  |
| Yes | 7 (13.7%) | 4 (7.8%) | 11 (10.8%) |
| No | 44 (86.3%) | 47 (92.2%) | 91 (89.2%) |
| **Bortezomib** |  |  |  |
| Yes | 48 (94.1%) | 49 (96.1%) | 97 (95.1%) |
| No | 3 (5.9%) | 2 (3.9%) | 5 (4.9%) |
| **Lenalidomide** | | | |
| Yes | 50 (98.0%) | 49 (96.1%) | 99 (97.1%) |
| No | 0 (0.0%) | 0 (0.0%) | 0 (0.0%) |
| Missing | 1 (2.0%) | 2 (3.9%) | 3 (2.9%) |
| **Total** | **51 (100%)** | **51 (100%)** | **102 (100%)** |

## 7. Progression free Survival

### 7.1. Hazard Ratios for progression-free survival using the Cox model, with corresponding 95% confidence intervals and P-values

| **Variable** | **Comparison** | **Hazard Ratio (HR)** | **Lower limit of 95% CI for HR** | **Upper limit of 95% CI for HR** | **P-value** |
| --- | --- | --- | --- | --- | --- |
| Treatment | CPD vs Pd | 0.70 | 0.44 | 1.11 | 0.1309 |
| Age | 60-69 vs <60 | 1.21 | 0.61 | 2.37 | 0.0706 |
|  | 70+ vs <60 | 0.67 | 0.33 | 1.33 |  |
| Prior lines of therapy | Greater than 3 vs 3 or less | 1.28 | 0.77 | 2.13 | 0.3457 |
| B2M | 3.5mg/L - 5.5mg/L vs Less than 3.5 mg/L | 2.60 | 1.42 | 4.75 | 0.0018 |
|  | Greater than or equal to 5.5mg/L vs Less than 3.5 mg/L | 2.44 | 1.31 | 4.53 |  |

## 8. Overall Survival

### 8.1. Parameter Estimates, standard errors and Hazard Ratios for overall survival using the Cox model, with corresponding 95% confidence intervals and P-values

| **Variable** | **Comparison** | **Estimate** | **SE** | **Hazard ratio (HR)** | **Lower limit of 95% CI for HR** | **Upper limit of 95% CI for HR** | **P-value** |
| --- | --- | --- | --- | --- | --- | --- | --- |
| Treatment | CPD vs Pd | 0.07 | 0.24 | 1.07 | 0.66 | 1.73 | 0.7816 |
| Age | 60-69 vs <60 | 0.50 | 0.39 | 1.64 | 0.76 | 3.55 | 0.2262 |
|  | 70+ vs <60 | 0.09 | 0.40 | 1.09 | 0.50 | 2.40 |  |
| Prior lines of therapy | Greater than 3 vs 3 or less | 0.45 | 0.26 | 1.57 | 0.94 | 2.61 | 0.0847 |
| B2M Result | 3.5mg/L - 5.5mg/L vs Less than 3.5 mg/L | 1.03 | 0.33 | 2.79 | 1.47 | 5.30 | 0.0003 |
|  | Greater than or equal to 5.5mg/L vs Less than 3.5 mg/L | 1.16 | 0.32 | 3.18 | 1.68 | 6.01 |  |

## 9. Treatment Response

### 9.1. Parameter estimates, standard errors and odds ratios of response to treatment, with corresponding 95% confidence intervals and p-values

| **Variable** | **Comparison** | **Estimate** | **SE** | **Odds Ratio** | **Lower 95% Confidence Limit for Odds Ratio** | **Upper 95% Confidence Limit for Odds Ratio** | **P-value** |
| --- | --- | --- | --- | --- | --- | --- | --- |
| Treatment | CPD vs Pd | 1.25 | 0.47 | 3.49 | 1.40 | 8.75 | 0.0056 |
| Age | 60-69 vs <60 | -0.25 | 0.66 | 0.78 | 0.21 | 2.85 | 0.0840 |
|  | 70+ vs <60 | 0.83 | 0.65 | 2.30 | 0.64 | 8.25 |  |
| Prior lines of therapy | 3 or less vs Greater than 3 | 1.28 | 0.52 | 3.59 | 1.29 | 10.00 | 0.0115 |
| B2M | 3.5mg/L - 5.5mg/L vs Less than 3.5 mg/L | -1.29 | 0.58 | 0.28 | 0.09 | 0.87 | 0.0728 |
|  | Greater than or equal to 5.5mg/L vs Less than 3.5 mg/L | -0.49 | 0.58 | 0.61 | 0.20 | 1.93 |  |

## 10. Safety

### 10.1. Number of participants with one or more SAE reported (mutually exclusive)

| **Has the patient had an SAE?** | **CPD (n=51)** | **Pd (n=51)** | **Total (n=102)** |
| --- | --- | --- | --- |
| Yes: SAR* | 42 (82.4%) | 31 (60.8%) | 73 (71.6%) |
| Yes: SAE | 2 (3.9%) | 7 (13.7%) | 9 (8.8%) |
| No | 7 (13.7%) | 13 (25.5%) | 20 (19.6%) |

*Patients experiencing a SAR who also experienced an SAE are included only in the SAR totals above.

### 10.2. Summary statistics of the number of SAEs reported

|  | **CPD** | **Pd** | **Total** |
| --- | --- | --- | --- |
| **Number of patients with one or more SAE** | 44 | 38 | 82 |
| **Number of SAEs reported** | 95 | 75 | 170 |
| **Number of SAEs per patient** |  |  |  |
| Mean (Standard Deviation) | 2.2 (1.78) | 2.0 (1.52) | 2.1 (1.65) |
| Median (Interquartile Range) | 1.0 (1, 3) | 1.5 (1, 2) | 1.0 (1, 3) |
| Range | (1, 9) | (1, 9) | (1, 9) |

### 10.3. Suspected relationship to trial medications for all reported SAEs

| **Is the event suspected to be related to the trial medications?** | **CPD N (%)** | **Pd N (%)** | **Total N (%)** |
| --- | --- | --- | --- |
| **Suspected to be related to the trial medications** | 77 (81.1) | 54 (72.0) | 131 (77.1) |
| - Suspected: Unlikely related to Pomalidomide, Dexamethasone | 0 (0.0) | 1 (1.9)* | 1 (0.8) |
| - Suspected: Dexamethasone | 5 (6.5) | 5 (9.3) | 10 (7.6) |
| - Suspected: Dexamethasone, Cyclophosphamide | 1 (1.3) | 0 (0.0) | 1 (0.8) |
| - Suspected: Pomalidomide | 7 (9.1) | 8 (14.8) | 15 (11.5) |
| - Suspected: Pomalidomide, Cyclophosphamide | 18 (23.4) | 0 (0.0) | 18 (13.7) |
| - Suspected: Pomalidomide, Dexamethasone | 2 (2.6) | 39 (72.2) | 41 (31.3) |
| - Suspected: Pomalidomide, Dexamethasone, Cyclophosphamide | 44 (57.1) | 1 (1.9) | 45 (34.4) |
| **Not suspected to be related to the trial medications** | 18 (18.9) | 21 (28.0) | 39 (22.9) |
| - Not suspected: No other cause** | 1 (5.6) | 1 (4.8) | 2 (5.1) |
| - Not suspected: Myeloma | 11 (61.1) | 11 (52.4) | 22 (56.4) |
| - Not suspected: Myeloma, Other illness*** | 0 (0.0) | 3 (14.3) | 3 (7.7) |
| - Not suspected: Other*** | 3 (16.7) | 4 (19.0) | 7 (17.9) |
| - Not suspected: Other illness*** | 3 (16.7) | 2 (9.5) | 5 (12.8) |
| **Total** | **95 (100.0)** | **75 (100.0)** | **170 (100.0)** |

*This SAE had attribution ‘Unlikely related’ for both Pomalidomide and Dexamethasone. As there is still some chance this SAE was related to trial medications it has therefore been classed as a SAR. **CPD arm: fall and Pd arm: breast cancer.

***14 SAEs not relating to trial medications were regarded as being related to ‘other’/’other illness’. The other causes for these were listed as “sun exposure” (for 2 SAEs), “reaction to platelet transfusion” and “diverticulosis” on the Pd arm and “anxiety”, “opiate analgaesia” and “Frailty, previous fall” on the CPD arm. The other illnesses reported with Myeloma were “recurrent diverticulitis”, “Br Asthma” and “COAD”. The other illnesses were “infection- unknown source” and “previously controlled AF” on the Pd arm and “parainfluenza”, “stroke” and “?Viral infrection” on the CPD arm.

### 10.4. All SAEs by MedDRA system organ class (currently mutually exclusive)

| **MedDRA System Organ Class** | **CPD N (%)** | **Pd N (%)** | **Total N (%)** |
| --- | --- | --- | --- |
| Blood and lymphatic system disorders | 6 (6.3) | 2 (2.7) | 8 (4.7) |
| Cardiac disorders | 1 (1.1) | 1 (1.3) | 2 (1.2) |
| Ear and labyrinth disorders | 1 (1.1) | 0 (0.0) | 1 (0.6) |
| Gastrointestinal disorders | 3 (3.2) | 4 (5.3) | 7 (4.1) |
| General disorders and administration site conditions | 1 (1.1) | 0 (0.0) | 1 (0.6) |
| Immune system disorders | 0 (0.0) | 1 (1.3) | 1 (0.6) |
| Infections and infestations | 63 (66.3) | 49 (65.3) | 112 (65.9) |
| Injury, poisoning and procedural complications | 0 (0.0) | 1 (1.3) | 1 (0.6) |
| Metabolism and nutrition disorders | 2 (2.1) | 2 (2.7) | 4 (2.4) |
| Musculoskeletal and connective tissue disorders | 4 (4.2) | 2 (2.7) | 6 (3.5) |
| Neoplasms benign, malignant and unspecified (including cysts and polyps) | 4 (4.2) | 5 (6.7) | 9 (5.3) |
| Nervous system disorders | 2 (2.1) | 1 (1.3) | 3 (1.8) |
| Renal and urinary disorders | 3 (3.2) | 2 (2.7) | 5 (2.9) |
| Respiratory, thoracic and mediastinal disorders | 2 (2.1) | 1 (1.3) | 3 (1.8) |
| Skin and subcutaneous tissue disorders | 2 (2.1) | 1 (1.3) | 3 (1.8) |
| Vascular disorders | 1 (1.1) | 3 (4.0) | 4 (2.4) |
| **Total** | **95 (100.0)** | **75 (100.0)** | **170 (100.0)** |

### 10.5. SAEs suspected to be related to trial medications by MedDRA system organ class

| **MedDRA System Organ Class** | **CPD N (%)** | **Pd N (%)** | **Total N (%)** |
| --- | --- | --- | --- |
| Blood and lymphatic system disorders | 6 (7.8) | 2 (3.7) | 8 (6.1) |
| Cardiac disorders | 1 (1.3) | 0 (0.0) | 1 (0.8) |
| Ear and labyrinth disorders | 1 (1.3) | 0 (0.0) | 1 (0.8) |
| Gastrointestinal disorders | 2 (2.6) | 3 (5.6) | 5 (3.8) |
| Infections and infestations | 55 (71.4) | 43 (79.6) | 98 (74.8) |
| Injury, poisoning and procedural complications | 0 (0.0) | 1 (1.9) | 1 (0.8) |
| Metabolism and nutrition disorders | 1 (1.3) | 1 (1.9) | 2 (1.5) |
| Musculoskeletal and connective tissue disorders | 1 (1.3) | 1 (1.9) | 2 (1.5) |
| Neoplasms benign, malignant and unspecified (including cysts and polyps) | 4 (5.2) | 0 (0.0) | 4 (3.1) |
| Nervous system disorders | 1 (1.3) | 1 (1.9) | 2 (1.5) |
| Renal and urinary disorders | 1 (1.3) | 0 (0.0) | 1 (0.8) |
| Respiratory, thoracic and mediastinal disorders | 1 (1.3) | 0 (0.0) | 1 (0.8) |
| Skin and subcutaneous tissue disorders | 2 (2.6) | 1 (1.9) | 3 (2.3) |
| Vascular disorders | 1 (1.3) | 1 (1.9) | 2 (1.5) |
| **Total** | **77 (100.0)** | **54 (100.0)** | **131 (100.0)** |

## 11. Adverse Events

### 11.1. Maximum grade AE experienced for each patient not reported as pre-defined term

| **Other Adverse event** | **CPD (n=51)** | **Pd (n=51)** | **Total (n=102)** |
| --- | --- | --- | --- |
| 0 | 5 (9.8%) | 7 (13.7%) | 12 (11.8%) |
| 1 | 7 (13.7%) | 10 (19.6%) | 17 (16.7%) |
| 2 | 15 (29.4%) | 16 (31.4%) | 31 (30.4%) |
| 3 | 22 (43.1%) | 14 (27.5%) | 36 (35.3%) |
| 4 | 1 (2.0%) | 4 (7.8%) | 5 (4.9%) |
| 5 | 1 (2.0%) | 0 (0.0%) | 1 (1.0%) |

Any ‘other’ AE that matched one of the pre-defined terms were included as that pre-defined term instead. 88.2% of patients reported some kind of ‘other’ adverse reaction. The most common ‘other’ adverse reactions were dyspnea (48 occurrences in 29 patients), white blood cell decreased (37 occurrences in 10 patients), fever (35 occurrences in 27 patients), lymphocyte count decreased (34 occurrences in 3 patients) and cough (31 occurrences in 25 patients). **Table below** presents all grade 3 and above ARs listed by treatment received and patient number, while all grade 4 and 5 other adverse reactions are highlighted.

### 11.2. Other Adverse Reactions (grade 3+) with grade 4 and 5 events highlighted

| **Population** | **Visit Cycle** | **Treatment or Follow-Up** | **Other CTCAE term** | **Other CTCAE Grade** |
| --- | --- | --- | --- | --- |
| CPD | 2 | Treatment | Sepsis | 3 |
| CPD | 8 | Treatment | Dyspnea | 3 |
| CPD | 11 | Treatment | Dyspnea | 3 |
| CPD | 7 | Treatment | Sepsis | 4 |
| CPD | 7 | Treatment | Sinus tachycardia | 3 |
| CPD | 9 | Treatment | Ear pain | 3 |
| CPD | 4 | Treatment | Fall | 3 |
| CPD | 1 | Treatment | Acute kidney injury | 3 |
| CPD | 1 | Follow-up | Fever | 3 |
| CPD | 1 | Follow-up | Dyspnea | 3 |
| CPD | 1 | Treatment | White blood cell decreased | 3 |
| CPD | 4 | Treatment | Lymphocyte count decreased | 3 |
| CPD | 15 | Treatment | Lymphocyte count decreased | 3 |
|  | 1 | Follow-up | Lymphocyte count decreased | 3 |
| CPD | 3 | Treatment | Cough | 3 |
| CPD | 13 | Treatment | Cataract | 3 |
| CPD | 16 | Treatment | Fever | 3 |
| CPD | 8 | Treatment | Anxiety | 3 |
| CPD | 3 | Treatment | White blood cell decreased | 3 |
| CPD | 5 | Treatment | White blood cell decreased | 3 |
| CPD | 1 | Treatment | Neck pain | 3 |
| CPD | 4 | Treatment | Malaise | 3 |
| CPD | 5 | Treatment | Syncope | 3 |
| CPD | 8 | Treatment | Lymphocyte count decreased | 3 |
| CPD | 9 | Treatment | Lymphocyte count decreased | 3 |
| CPD | 9 | Treatment | White blood cell decreased | 3 |
| CPD | 11 | Treatment | Fracture | 3 |
| CPD | 11 | Treatment | White blood cell decreased | 3 |
| CPD | 6 | Treatment | Sepsis | 3 |
| CPD | 6 | Treatment | Syncope | 3 |
| CPD | 11 | Treatment | Neoplasms benign, malignant and unspecified (incl cysts and polyps) - Other, specify | 5 |
| CPD | 9 | Treatment | Hyperglycemia | 3 |
| CPD | 13 | Treatment | Syncope | 3 |
| CPD | 2 | Treatment | Confusion | 3 |
| CPD | 1 | Treatment | Lymphocyte count decreased | 3 |
| CPD | 9 | Treatment | Fever | 3 |
| CPD | 1 | Treatment | Fever | 3 |
| CPD | 3 | Treatment | Dyspnea | 3 |
| CPD | 1 | Treatment | Sepsis | 3 |
| CPD | 1 | Treatment | Fever | 3 |
| CPD | 3 | Treatment | Acute kidney injury | 3 |
| CPD | 3 | Treatment | Nervous system disorders - Other, specify | 3 |
| CPD | 1 | Treatment | Fever | 3 |
| CPD | 2 | Treatment | Abdominal distension | 3 |
| CPD | 2 | Treatment | Hypotension | 3 |
| Pd | 1 | Treatment | Dyspnea | 3 |
| Pd | 1 | Treatment | Sepsis | 4 |
| Pd | 3 | Treatment | Epistaxis | 3 |
| Pd | 3 | Treatment | Gastric ulcer | 3 |
| Pd | 3 | Treatment | Upper gastrointestinal hemorrhage | 3 |
| Pd | 1 | Treatment | Sepsis | 3 |
| Pd | 1 | Treatment | Intracranial hemorrhage | 4 |
| Pd | 6 | Treatment | Headache | 3 |
| Pd | 1 | Treatment | Cataract | 3 |
| Pd | 1 | Treatment | Confusion | 3 |
| Pd | 1 | Treatment | Flank pain | 3 |
| Pd | 1 | Treatment | Back pain | 3 |
| Pd | 1 | Treatment | Sepsis | 4 |
| Pd | 2 | Treatment | Confusion | 3 |
| Pd | 5 | Treatment | Cough | 3 |
| Pd | 5 | Treatment | Dyspnea | 3 |
| Pd | 1 | Treatment | Sepsis | 4 |
| Pd | 4 | Treatment | White blood cell decreased | 3 |
| Pd | 5 | Treatment | Fall | 3 |
| Pd | 12 | Treatment | Dyspnea | 3 |
| Pd | 2 | Treatment | Abdominal pain | 3 |
| Pd | 2 | Treatment | Acidosis | 3 |
| Pd | 2 | Treatment | Hyperglycemia | 3 |
| Pd | 2 | Treatment | Muscle weakness lower limb | 3 |
| Pd | 4 | Treatment | Hyperglycemia | 3 |
| Pd | 1 | Treatment | White blood cell decreased | 3 |
| Pd | 2 | Treatment | Dehydration | 3 |
| Pd | 2 | Treatment | Hypokalemia | 3 |
| Pd | 2 | Treatment | Hypotension | 3 |
| Pd | 2 | Treatment | White blood cell decreased | 3 |
| Pd | 4 | Treatment | Anorexia | 3 |
| Pd | 4 | Treatment | White blood cell decreased | 3 |
| Pd | 5 | Treatment | Fall | 3 |
| Pd | 1 | Treatment | Hypertension | 3 |
| Pd | 1 | Treatment | Insomnia | 3 |
| Pd | 1 | Follow-up | Back pain | 3 |
| Pd | 1 | Follow-up | Flank pain | 3 |
| Pd | 1 | Follow-up | Pain in extremity | 3 |

## 12. Deliverability

In total, there were 215 cycles of treatment detailed as being delayed, in 72 patients (37 CPD, 35 Pd). Toxicity accounted for 34.7% of CPD cycle delays and 30.6% of Pd cycle delays.

In total, there were 56 patients with at least one dose reduction to any drug (35 CPd, 21 Pd). On the CPd arm, the most common reason for dose reductions to cyclophosphamide and pomalidomide was hematological toxicity (84.3% of cyclophosphamide reductions and 61.5% of pomalidomide reductions) while the most common reason for dose reductions to dexamethasone was non-hematological toxicity (64.7%) (**Table Below**). On the Pd arm, the most common reason for dose reductions to both drugs were non-hematological toxicity (61.5% of pomalidomide reductions and 75.0% of dexamethasone reductions). More pomalidomide reductions were seen in patients on the CPd arm compared to the Pd arm (18 vs 10) while the number of patients with dexamethasone reductions was similar across arms (15 vs 14).

In total there were 80 patients with at least one dose omission of any drug (44 CPd, 36 Pd). On the CPd arm, the most common reason for dose omissions of pomalidomide, cyclophosphamide and dexamethasone were hematological toxicity (50.7% of pomalidomide omissions, 61.5% of cyclophosphamide omissions and 43.1% of dexamethasone omissions). On the Pd arm, the most common reason for dose omissions of both drugs was due to non-hematological toxicity (48.1% of pomalidomide omissions and 41.7% of dexamethasone omissions). More patients had pomalidomide and dexamethasone omissions on the CPD arm compared to the Pd arm with 44 pomalidomide omissions on the CPD arm vs 36 omissions on the Pd arm and 36 patients with at least one dexamethasone omissions on the CPD arm vs 32 omissions on the Pd arm.

Across both arms, mean doses for dexamethasone (CPd 29.3mg, Pd 26.1mg) and pomalidomide (CPd 3.5mg, Pd 3.6mg) were similar. The mean dose of cyclophosphamide was reduced from 500mg to 407.8mg, reflecting the preferential order of dose reductions on the CPd arm.

### 12.1. Dose modifications.

| **Dose modifications** | **CPD (n=51)** | **Pd (n=51)** |
| --- | --- | --- |
| ***Pomalidomide*** |  |  |
| Patients with dose reductions | 18 (35.3%) | 10 (19.6%) |
| Patients with dose omissions | 44 (86.3%) | 36 (70.6%) |
| **Reasons Dose Reduced** |  |  |
| Haematological toxicity | 16 (61.5%) | 3 (23.1%) |
| Non-haematological toxicity | 8 (30.8%) | 8 (61.5%) |
| Other | 1 (3.8%) | 2 (15.4%) |
| Missing | 1 (3.8%) | 0 |
| Total | 26 (100%) | 13 (100%) |
| **Reasons Dose Omitted** |  |  |
| Haematological toxicity | 34 (50.7%) | 10 (19.2%) |
| Non-haematological toxicity | 21 (31.3%) | 25 (48.1%) |
| Logistical | 0 | 1 (1.9%) |
| Other | 12 (17.9%) | 15 (28.8%) |
| Missing | 0 | 1 (1.9%) |
| Total | 67 (100%) | 52 (100%) |
| ***Dexamethasone*** |  |  |
| Patients with dose reductions | 15 (29.4%) | 14 (27.5%) |
| Patients with dose omissions | 36 (70.6%) | 32 (62.7%) |
| **Reasons Dose Reduced** |  |  |
| Haematological toxicity | 2 (11.8%) | 1 (6.3%) |
| Non-haematological toxicity | 11 (64.7%) | 12 (75.0%) |
| Logistical reason | 1 (5.9%) | 0 |
| Other | 3 (17.6%) | 3 (18.8%) |
| Total | 17 (100%) | 16 (100%) |
| **Reasons Dose Omitted** |  |  |
| Haematological toxicity | 25 (43.1%) | 10 (20.8%) |
| Non-haematological toxicity | 22 (37.9%) | 20 (41.7%) |
| Other | 11 (19.0%) | 18 (37.5%) |
| Total | 58 (100%) | 48 (100%) |
| ***Cyclophosphamide*** |  |  |
| Patients with dose reductions | 28 (54.9%) | NA |
| Patients with dose omissions | 29 (56.9%) | NA |
| **Reasons Dose Reduced** |  |  |
| Haematological toxicity | 43 (84.3%) | NA |
| Non-haematological toxicity | 4 (7.8%) | NA |
| Other | 4 (7.8%) | NA |
| Total | 51 (100%) | NA |
| **Dose Omitted** |  |  |
| Haematological toxicity | 32 (61.5%) | NA |
| Non-haematological toxicity | 12 (23.1%) | NA |
| Logistical reason | 1 (1.9%) | NA |
| Other | 7 (13.5%) | NA |
| Total | 52 (100%) | NA |

## 13. Treatment specific effects on T-cell subsets

### 13.1. Total T cells

### 13.2. CD4+ T cells

### 13.3. CD8+ T cells

### 13.4. Proliferating T cells

### 13.5. Proliferating CD4+ T cells

### 13.6. Proliferating CD8+ T cells (include)

### 13.7. Naïve or effector CD4+ T cells

### 13.8. Naïve CD4+ T cells

### 13.9. Effector CD4+ T cells

### 13.10. Central memory CD4+ T cells

### 13.11. Effector memory CD4+ T cells

### 13.12. Naïve or effector CD8 + T cells

### 13.13. Naïve CD8+ T cells

### 13.14. Effector CD8+ T cells

### 13.15. Central memory CD8+ T cells

### 13.16. Effector memory CD8+ T cells

### 13.17. Proliferating naïve or effector CD4+ T cells

### 13.18. Proliferating naïve CD4+ T cells

### 13.19. Proliferating effector CD4+ T cells

### 13.20. Proliferating central memory CD4+ T cells

### 13.21. Proliferating effector memory CD4+ T cells

### 13.22. Proliferating naïve or effector CD8+ T cells

### 13.23. Proliferating naïve CD8+ T cells

### 13.24. Proliferating effector CD8+ T cells

### 13.25. Proliferating central memory CD8+ T cells

### 13.26. Proliferating effector memory CD8+ T cells

### 13.27. Activated CD4+ T cells

### 13.28. Activated CD8+ T cells

### 13.29. Proliferating activated CD8+ T cells

### 13.30. Proliferating activated CD4+ T cells

### 13.31. Ratio CD4+:CD8+ T cells

## 14. Pre-treatment T-cells and their effect on response and prognosis

### 14.1. Total T cells (C1D1) response analysis

### 14.2. CD4+ T cells (C1D1) Response analysis

### 14.3. CD8+ T cells (C1D1) Response analysis

### 14.4. Effector memory CD8+ T cells (C1D1) Response analysis

### 14.5. Activated CD8+ T cells (C1D1) Response analysis

### 14.6. CD4+ T cells Prognostic PFS analysis

Likelihood ratio test for non-parametric prognostic model (p=0.02).

Plot of parametric prognostic model:

Cox PH model including term for parametric CD4+ term.
